# Which primary care model? A qualitative analysis of ward-based outreach teams in South Africa

**DOI:** 10.4102/phcfm.v9i1.1252

**Published:** 2017-05-31

**Authors:** Tessa S. Marcus, Jannie Hugo, Champak C. Jinabhai

**Affiliations:** 1 Department of Family Medicine, School of Medicine, University of Pretoria, South Africa; 2 School of Health Sciences, University of Fort Hare, South Africa

## Abstract

**Abstract:**

Globally, models of extending universal health coverage through primary care are influenced by country-specific systems of health care and disease management. In 2015 a rapid assessment of the ward-based outreach component of primary care reengineering was commissioned to understand implementation and rollout challenges.

**Aim:**

This article aims to describe middle- and lower-level managers’ understanding of ward-based outreach teams (WBOTs) and the problems of authority, jurisdiction and practical functioning that arise from the way the model is constructed and has been operationalised.

**Setting:**

Data are drawn from a rapid assessment of National Health Insurance (NHI) pilot sites in seven provinces.

**Methods:**

The study used a modified version of CASCADE. Peer-review teams of public health researchers and district/sub-district managers collected data in two sites per province between March and July 2015.

**Results:**

Respondents unequivocally support the strategy to extend primary health care services to people in their homes and communities both because it is responsive to the family context of individual health and because it reaches marginal people. They, however, identify critical issues that arise from basing WBOTs in facilities, including unspecific team leadership, inadequate supervision, poorly constituted teams, limited community reach and serious infrastructural and material under-provision.

**Conclusion:**

Many of the shortcomings of a facility-based extension model can be addressed by an independently resourced, geographic, community-based model of fully constituted teams that are clinically and organisationally supported in an integrated district health system. However, a community-oriented primary care approach will still have to grapple with overarching framework problems.

## Introduction

Community-based primary health care is increasingly recognised as an essential part of a strong health care delivery system.^1^

It is seen as a sustainable and affordable way of achieving public health goals across all segments of society.^2^ While efforts to achieve universal health coverage through primary care all involve community health workers (CHWs) of some description, actual intervention models around the globe vary quite considerably.^3^ Not only are they place and time specific, but they are also strongly influenced by historical and contemporary country-specific practices and their embedded assumptions. This is true for South Africa.

National health care system reform was initiated by the South African government in 2011.^4^ It includes community-based outreach services, school health services, effective referral systems and improved emergency and planned patient transport services delivered through purpose-built teams. The intention is that district specialist teams improve facility functioning, and the school health and ward teams extend existing facility services through outreach to schools and communities.^5^ In this model these and all other tiers and divisions of health care provision are to be integrated through a National Health Insurance (NHI) financing system.

The ward-based outreach component of primary care reengineering was initiated in nine NHI pilot sites. In 2015, three years into implementation, the National Department of Health commissioned a rapid assessment of ward-based outreach team (WBOT) rollout in seven provinces, in order to understand reform challenges and to identify ways of developing and scaling the approach beyond the NHI pilot sites.

This article presents findings on middle- and lower-level managers’ (district and sub-district managers, facility managers and WBOT leaders) understanding of the purpose and approach to WBOTs and the problems of authority, jurisdiction and practical functioning that arise from the way the model is constructed and has been operationalised.

## Research methods and design

The rapid assessment used a modified version of CASCADE,^6^ a qualitative peer-review methodology developed by the European Union-funded Cities Exchanging on Local Energy Leadership programme. It is designed to learn from and strengthen sites and systems by promoting equity in learning and assessment.

Research for this study was undertaken in two sub-districts in each NHI pilot district in seven provinces according to a prescribed work plan (Box 1). At the request of the National Department of Health (NDoH), three ‘best practice’ initiatives (City of Tshwane/UP COPC Intervention; Philani Maternal Child Health and Nutrition Project; and Community Action and Palliative Care) were included as case studies in the rapid assessment. They were either not in an NHI pilot site or they were run by the local municipality in an NHI pilot site but not included in the assessment’s terms of reference.

BOX 1 WBOT Rapid Assessment Workplan.

Day 1: Arrival and initial management meetings (an overview of the study objectives and the research process and ensuring familiarity with the benchmark self-assessment);

Day 2 & 3: Interviews and observations – with each field worker allocated to between one and three WBOTs;

Day 4: Researcher meetings and debriefing session to compile a draft report;

Day 5: Peer learning workshop to share and reflect with management and other key stakeholders on the emerging review.

*Source*: Jinabhai et al.^7^

Study sites were purposively selected with the assistance of district health staff. The best and the worst performing sub-district in respect of primary care reengineering were included in the research.

Two research teams (one from each of the participating universities) undertook field research, visiting each site in their designated provinces. Each team comprised two to five university researchers and two or more health care managers, professionals and/or workers selected from study sites. Research teams spent five days in each assigned sub-district.

Data were collected using a purposively designed set of ‘benchmarks for integrated learning’ that guided all fieldwork conducted by the research teams. The benchmarks focused on five key topics – mindset/approach, preparation, implementation, networking/relationships and monitoring/evaluation. These topics were used to design a pre-visit questionnaire. The benchmarks and sub-questions were also used to guide in field observations, key informant interviews and focus group discussions at each site.

The questionnaire was distributed to district and sub-district managers as well as team leaders prior to fieldwork. It was completed by respondents and collected during site visits. The findings presented here are based on an analysis of questions in the first three benchmark topics that relate to the article’s focus – drawing either from responses in the questionnaire (for the NHI sites) or in respect of the case study, from document reviews and key informant interviews. A total of 44 questionnaires were returned and four key informant interviews conducted.

Questionnaire responses were captured into MS Excel. The benchmarks and questions provided a clear framework for analysis. The authors intensively read and discussed the data, leading to an identification of themes, common issues and exceptions. Quotations best illustrative of the evidence for the arguments are provided in the next section.

## Ethical consideration

Ethical approval to conduct this study was obtained from the research ethics committees of the University of Fort Hare (REC-270710-028-RALevel01) and the University of Pretoria (Ethics Reference Number: 102/2011 Amendment 26062014).

## Results

### Approach and purpose

In terms of the approach and purpose of ward-based health care, respondents across most study districts understand the national model as one of clinic-based, primary health care service extension.

‘[*WBOTs are*] the eyes, ears and hands of the clinic sisters.… WBOT is there to help the sister in the PHC setup to reach the patient to oversee the adherence of the patient treatment.’ (Northern Cape, SA RES2, Female; SA RES6, Female)

‘This is an extra hand to the fixed facilities. Previously PHC nurses were solely responsible to reach out to the communities. WBOTs are expected to deliver PHC services to communities. They are attached to fixed health facilities and operate there.’ (Free State, SA RES6, Female)

‘The ward-based outreach team serves as the bridge between the community and health facilities.’ (Limpopo, SA RES3, Female)

In the Eastern Cape and City of Tshwane (Gauteng), WBOTs are not clinic based, although all teams are linked to defined clinics. In the City of Tshwane, they are based in and run from health posts situated at pre-existing, available local service sites, such as schools, non-governmental organisation (NGO) offices or churches as well as hospitals and clinics. In Eastern Cape respondents describe operating from ‘one stop’ health service points in communities.

‘There are service points where we do all the programs at least once a month together with NGOs [*HIV, AIDS and TB*].’ (Eastern Cape, SA RES4, Female)

Respondents distinguish the WBOT approach from the legacy model of facility-based primary health care service, which they describe as follows:

‘Primary health care was normally seen as the entry level of service that the community accessed through a primary health care facility. Patients had to come to the facility to get a service. District services from clinic staff to households were extremely limited and services therefore were mainly geared at patients coming to the facility.’ (Northern Cape, SA RES5, Female)

In the WBOT model, by contrast, respondents say teams are expected to work in defined areas with defined people and communities.

‘WBOT differs from PHC in that the WBOT identifies a catchment area for each CHW. WBOT allocates households to community health workers.’ (Eastern Cape, SA RES8, Female)

They should extend health care beyond the individual patient.

‘While in primary health care [*facilities*] the focus is on addressing the problems that a patient presents with, but not extending to family health, which in most cases it is the cause of ill-health. WBOT is community, family and individual orientated. (Limpopo, SA RES1, Female)

‘Primary health care used to go to specific patients not covering the whole catchment area.’ (Free State, SA RES4, Female)

Health care teams are expected to provide services to people in their homes.

‘WBOTs are different from PHCs because services are provided at the household. The source of the problem is seen in the household. If there is diarrhea, when visiting, you find unhygienic methods … contaminated water.’ (Eastern Cape, SA RES5, Female)

‘With WBOTs the entry level of the service is at community/household level.… WBOTs see the person’s needs at home – my eyes and hands in community. The WBOT knows exactly the health profile of the family in that household. They can give health information to the family themselves; refer to the clinic or follow-up referrals from the health staff at facility level….’ (Northern Cape, SA RES5, Female)

WBOTs are expected to make it possible to extend health care to people who are on the social or physical margins of the health care system.

In Tshwane Inner City, for instance, in addition to going to households, WBOTs go out to people who are homeless, abuse substances, engage in sex work or are forced to the fringe of society in other ways (Dr Mohale M 2015, in-depth interview, August 28).

They also aim to reach rural and remote communities.

‘In primary health care people were defaulting treatment because of bad roads and the long distances they used to have to travel. The WBOT team is able to visit households in the community as well as screen children in schools.’ (Eastern Cape, SA RES3, Female)

### Team composition and system functioning

Respondents say the model is one in which services are delivered by teams made up of:

‘One professional nurse, six or more CHWs, one health promoter and one environmental officer.’ (Mpumalanga, SA RES2, Female)

and that WBOT personnel will be recruited into or deployed from within the health system and government-funded NGOs.

In terms of operationalising WBOTs according to the NDoH model, respondents describe model-related challenges of functional authority and jurisdiction as well as implementation challenges of system preparation and understanding, human resourcing, infrastructure and materials.

Respondents report that WBOTs are poorly understood at various levels of management in the public health care system.

‘[*There is a*] lack of knowledge on the part of management on WBOT activities and roles leading to restrictive behaviours on the part of some local area managers.’ (Free State, SA RES3, Female)

Respondents report that they have little control over their working days.

‘It is not possible for me to plan my duties since I never know where I will be and when somebody who is an operational manager is planning about me.’ (Mpumalanga, SA RES3, Female)

They find that there is a similar lack of understanding of the model where CHWs are employed by NGOs, including those funded by provincial government.

‘Currently the WBOTs still form part of Hospice and get their stipend through Hospice. The stipends are paid over to Hospice from Department of Health from the HIV grant.… WBOTs being appointed by an NGO [*Hospice*] have to account to Hospice AND be part of DoH. It is difficult to manage working hours, responsibilities and data for NGO and DHIS (District Health Information System).’ (Northern Cape, SA RES5, Female; SA RES8, Female)

‘CHW’s were recruited from funded NGOs and were trained as CHWs, but they are still receiving stipends from their respective NGOs.… NGO managers have a different understanding regarding the role of CHWs in WBOT. CHWs are not allowed to go out and perform WBOT. They are told to do that during the weekend in their own time.’ (Limpopo, SA RES1, Female; SA RES2, Female)

In terms of human resourcing all study sites experience common interrelated issues. WBOTs are supposed to be led by professional nurses.

Health system managers most often assign facility-based professional nurses as WBOT team leaders.

‘Team leaders were identified from staff from the facility and training was given to them.… Dedicated team leaders are not yet appointed.’ (Northern Cape, SA RES5, Female)

‘Operational managers in clinics identified team leaders…. Team leaders were recruited from non-busy clinics and are professional nurses attached to primary health care structure.’ (Limpopo, SA RES2, Female)

‘A professional nurse was delegated from the facility.’ (Mpumalanga, SA RES2, Female)

‘[*Initially*] the team leader was allocated from other contract programmes – HCT [*HIV Counselling and Testing*] and TB [*tuberculosis*]. Later a permanent appointment of an OTL [*Outreach Team Leader*] was done…’ (Free State, SA RES3, Female)

Or they appoint enrolled nurses as team leaders.

‘Originally team leaders were supposed to be PNs [*professional nurses*]. In our district they now allocate ENs [*enrolled nurses*] to OTLs.’ (Free State, SA RES1, Female)

In part they take this route because recruitment into the system is stymied by a dearth of professional nurses.

‘In my district 53 posts for OTLs [*Outreach Team Leaders*] were advertised and only 10 filled.’ (Free State, SA RES6, Female)

In some sites management has recruited retired professional nurses to get around the shortage of skills.

‘Many team leaders recruited by the City of Tshwane are retired professional nurses.’ (Gauteng, SA RES6, Female)

Respondents observe that the approach of locating WBOTs in and under the supervision of primary health care facilities overburdens facility management.

‘Our facilities are understaffed even before the WBOT. [*It*] is adding a serious strain to our facility managers as the facility work is still as it was, if not worse, but team leaders are required to do home visits, attend meetings and workshops and the facilities are balancing their shifts with WBOT teams.’ (Limpopo, SA RES1, Female)

‘Most WBOTs do not have team leaders so the clinic manager of their parent clinic acts as their team leader. In such cases the clinic would not be able to provide adequate supervision.’ (Free State, SA RES5, Female)

This approach also is onerous on CHWs who lose valuable working time reporting before and after work at their base clinics.

‘Time is wasted between homes and catchment clinics.’ (Free State, SA RES3, Female)

And it negatively affects the functioning of WBOTs.

‘Management in the programme is on and off.’ (Eastern Cape, SA RES3, Female)

‘At present the team leader is part of the staff of the clinic. It is very difficult to have a team leader that has to work at the clinic as well.… [*She*] does not have the time for WBOTs.’ (Northern Cape, SA RES7, Female)

‘Education and training is difficult, because I’m full time working in a clinic.’ (Northern Cape, SA RES6, Female)

‘WBOT is taking a back seat in our facilities’ priorities. Team leaders are full-time nurses at their respective clinics. It is hard to do home visits as required by WBOT.’ (Limpopo, SA RES1, Female)

‘I am not able to manage the team properly since some of the days I have to work inside the clinic.’ (Mpumalanga, SA RES3, Female)

‘Some team leaders are kept in the clinic.’ (Gauteng, SA RES6, Female)

As a consequence of the shortage of team leaders, in several districts respondents report that team leaders are required to manage more than one team.

‘A registered nurse that was a TB tracer was used to start reengineering. She was doing both these duties as well as managing six CHWs…’ (Free State, SA RES1, Female)

‘I first had to lead two teams for two months.… After another team leader was appointed I started managing my team.’ (North West, SA RES3, Female)

They also point out that there are CHWs without team leaders.

‘Other team leaders are contract workers, so for about two to three months the ward is without a team leader.’ (Eastern Cape, SA RES4, Female)

‘Most WBOTs do not have team leaders so the clinic manager of their parent clinic acts as their team leader. In such cases the clinic would not be able to provide adequate supervision.’ (Free State, SA RES5, Female)

Some observe that team leaders based at facilities are remote and distant from the teams they supervise.

‘[*It is*] difficult for them to report weekly to the team leader at the facility because of the distance and [*it is*] difficult for team leader to do supervision because of transport.’ (Eastern Cape, SA RES4, Female)

In addition to team leadership challenges, respondents also report problems with team composition. WBOTs are supposed to be made up of at least eight community health and home-based care workers. Several respondents report that there are either too few or too many CHWs in teams. They say that this affects both the ability of WBOTs to provide services and the ability of team leaders to properly manage their teams.

‘The WBOT comprises four CHWs. [*It*] is allocated to one ward. The challenge is that when performing their duties they have to cover a ward population that is far larger than the required numbers.’ (Free State, SA RES8, Female)

‘There is a shortage of CHWs. CHWs serve three wards and above.… [*The*] shortage of CHWs is causing desperation.… Other areas have no CHWs at all so no information is collected, no challenges are met or solved.’ (Eastern Cape, SA RES1, Female; SA RES2, Female; SA RES7 Female)

‘For effective implementation, monitoring and evaluation, at least each team leader [*should*] have 12 CHWs, not large numbers.’ (Gauteng SA RES1 Female)

There are also gaps in team composition. Respondents’ appeal for the appointment of environmental health officers and health promoters to local areas ‘if it cannot be afforded per ward’ (Free State) and that ‘fully formed teams be established including specialists and environmental health practitioners’ (Limpopo) suggests that these envisaged appointments have not been made in many districts, with the exception of parts of the Eastern Cape. Several respondents also point to the need for social workers to be integrated into the programme (North West, Limpopo).

### Infrastructure and resourcing

In all provinces respondents say that attention to infrastructure and material resourcing of ward-based primary health services is inadequate. They say that NHI grants are both restrictive and subject to competing interests.

‘There is no clear strategy from a provincial level – no dedicated budget to implement. [*We*] use the NHI grant for certain aspects, but it is very prescriptive and cannot be used for anything.’ (Northern Cape SA RES5 Female)

As a consequence, districts and facilities have had to devise solutions from within already limited means.

‘There was a lot of pressure on the district to start the service. The district therefore had to work out its own strategy.’ (Northern Cape, SA RES5, Female)

They describe how these constraints seriously hamper their ability to work in a multiplicity of ways. All respondents report problems of space.

‘Where do you start if you hardly have a place to work from? How do you organise and allocate work? Reporting back from field needs a place.… There is no space for the teams. Most of the time you will be told that reengineering doesn’t have a budget for offices. Sometimes we are outside the facility.’ (North West, SA RES1, Female; SA RES4, Female)

‘Prepare space for the teams to work. It is a problem now that the team is working in a very small clinic that does not accommodate workers.’ (Free State, SA RES6, Female)

‘Infrastructure at some of the clinics is very poor, because the clinic is too small.’ (Northern Cape, SA RES2, Female)

Respondents report a general shortage of essential office equipment to support WBOT functioning, including not having desks and chairs, lockable filing cabinets, photocopying equipment and stationery to register and collect information, where the system is paper based, and access to phones and computers.

‘At some of the clinics there are no fax or photocopy machine or the machines are faulty or out of order.’ (Northern Cape, SA RES2, Female)

‘Initially they were provided with cell phones but now they are not functional. So they can’t communicate risks which need the OTL intervention.’ (Free State, SA RES8, Female)

‘[*There is*] no furniture for our offices. We are not able to communicate with CHWs because there are no phones.’ (Eastern Cape, SA RES3, Female)

‘There is no space to put or store daily information collected from households, therefore we cannot maintain confidentially of household members.’ (Gauteng, SA RES5, Female)

Respondents also point to a lack of uniforms, name tags that identify CHWs to the community, protective clothing, properly equipped kitbags and essential equipment.

‘The ward is deep rural, walking distances are long. [*CHWs work*] with no protective clothing.… Adequate medical equipment is needed – BP machines [*portable one*], HB machines and a bag to carry the stuff. We also need to be provided with duster coats, takkies, umbrellas and sun hats.’ (Eastern Cape, SA RES2, Female; SA RES7, Female)

‘You want to get up and want to go to work, but sometimes it’s wet. (North West, SA RES4, Female)

Respondents report that their work is severely affected by transport constraints.

‘Ferrying CHWs to search out their destinations is a problem. They do not have access to transport … [*because*] transport is unavailable we postpone appointments with clients at service points.’ (Eastern Cape, SA RES2, Female)

‘We were promised cars when we started. I drive a small bakkie. I can only take one CHW at the time otherwise they should sit at the back.… I travel 25–27 km from work, 58 km daily. Claiming money for our travelling is a nightmare. Our forms are always returned for small mistakes.’ (North West, SA RES4, Female)

### The strategy and the model

Among respondents there is unequivocal support for the strategy of taking health care to people in their homes.

‘It’s the best thing ever to happen to our country. Not only are we addressing the health of our people, but most importantly, we are entering their household to identify the possible causes of [*disease*] and by assessing the community as a whole.’ (Limpopo, SA RES1, Female)

‘The difference that I see is the fact that for primary health the client has to come to the facility or mobile clinic to access health care, whereas in WBOTs the client gets health care at his household. This service is an economic relief for them especially for disadvantaged communities that are very far from health care.’ (Eastern Cape, SA RES8, Female)

However, from their experiences of the challenges of the model and its practical implementation they make five key recommendations that relate to the findings presented in this article. They suggest that ward-based health care should be

- part of an integrated health care system.
- located outside of clinics.
- budgeted for independently.
- staffed to meet service needs (teams with team leaders, professionally supported).
- equipped to be functional and effective.

## Discussion

In 2010 the National Department of Health^8^ introduced primary care reengineering as part of a multi-pronged system reform. In it WBOTs were initiated as a way of extending the reach of existing facilities at the bottom of the health care hierarchy. As the nomenclature ‘outreach’ suggests, the model is one of creating WBOTs as an add-on service attached to and managed by primary care clinics. With the exception of Tshwane, this is in fact how the model has been practically implemented in all districts.

The extension of facility services model is not unique to South Africa,^9^ nor is it unique to the contemporary period.

It also is neither the only possible nor the most appropriate response to the health gap in contemporary South Africa. The ‘unsteady march’ to improve the health of ordinary people through community-focused health promotion and disease prevention dates back to the community health centre movement first advocated for (UK and USA) and implemented (Soviet Union, Ceylon/Sri-Lanka and China) in the 1920s^10,11^ and subsequently expanded and elaborated into community-oriented primary care (COPC) by the Karks in the 1940s in South Africa.^12^ Building on an initial Tshwane District (Gauteng Province) pilot (2011–2013), the City of Tshwane, together with the University of Pretoria, has used COPC as an approach to build a community health platform. In it ward health teams are based in health posts and linked to clinics and other district health facilities as well as private sector practices.^13^

There are several insurmountable problems that flow from the facility-based extension model. In the first instance there is the difficulty of its physically specific but structurally tangential location in the health care system. By government’s own account primary care reengineering was initiated to address the extreme pressures that ill-health places on health care facilities and national well-being in general.^14^ It was also introduced to achieve universal health coverage that the existing facility-based, specialist-focused and disease-driven system is unable to address.

As respondents describe, attaching WBOTs to clinics adds additional management and service responsibilities onto already strained, overstretched, under-resourced and underperforming clinics and CHWs.^15,16,17^

The model creates functional tensions within the health care system. Team leaders who work within facilities have to juggle the demands of their clinic based responsibilities with the expectation that they simultaneously work with CHWs in the wards. Inevitably, they are inclined to prioritise their facility work at the expense of community health-based activities. It also creates functional tensions between the health care system and the NGOs that formally employ CHWs. Because CHWs work *under* the clinics but *for* NGOs, team leaders (and CHWs) have to manage conflicts of authority and jurisdiction. They also have to manage conflicting organisational cultures because in the NGO sector, CHWs frequently work with little or no supervision, whereas in the public health care system there is both a clear hierarchy of authority and an expectation of supervision and oversight.

The model is unable to address the vexing issue of clinical leadership and oversight that is essential to the quality and impact of all health care services and is integral to the intent of universal health coverage.^18^ As described, the difficulty of recruiting professional nurses to lead teams and the expectation that those employed in clinics also work in the communities inevitably short-change ward health services and leave CHWs under- or unsupported. Despite government’s best intentions, within the existing human resource development framework it is impossible to circumvent the absolute shortage of skilled professional health workers. Like many countries around the world, South Africa faces a growing crisis in human resources for health.^19^ It is this crisis that is fuelling a global drive to fundamentally reform national and international approaches to capacity development.^20^ Many of these issues relate to who constitutes the health care workforce as well as the education and training they require to be able to deliver quality services. In addition, there are organisational dimensions of the model of delivery that also need to be reformed. One solution that has been proposed (CDC 2016, personal communication, October 6) is to create functional health catchment areas in each district to drive clinician-led inter-professional care in the community health platform. Defined by population, physical geography and existing facility services, each health catchment area should have a team of locally available health and care professionals led by a clinician (a family physician or general practitioner or clinical associate) and should involve both a professional nurse and a social worker. It can also include other professional practitioners (e.g. psychologists, occupational, physical, audio and speech therapists, environmental health officers, nutritionists), depending on their availability.^21^ This solution draws on the experiences of implementing COPC with the City of Tshwane and is in keeping with the establishment of district specialist teams to support the facility platform that is part of primary care reengineering.

The problem of insufficient planning, especially around financing and the consequent inadequate material resourcing of WBOTs is related in part to the overall framework and in part to the model. It could have arisen from an assumption at national and provincial level that dedicated financing was not necessary as additional resources would be met from NHI and other grants. This has not happened. Notwithstanding provincial variations^2^ it also may be a consequence of inadequate preparation of and engagement with district and facility health care managers and professionals. Respondents mentioned both these possibilities. Other reasons also may be behind the poor planning and resourcing of WBOTs. As in many other countries^1^ it might be because of the unfounded assumption that ward-based health care could be implemented at a low cost and within the existing budgets by rearranging personnel, space, equipment and time at the facility. Faced with system expectations and under-resourcing, in practice, this is precisely what clinic and district managers say they have done. They organise and run WBOTs around facility services and needs. As laudable as their pragmatic response is, however, it is unlikely to yield the expected or potential health and economic returns. Generally, the evidence from South Africa^22^ and other middle- and low-income countries is that a community-based health platform involving CHWs requires dedicated upfront and recurrent national funding.^1^

The facility-based model is unable to adequately address the equity issues that underpin the drive for universal health coverage. Its design and organisation, including having too few teams, having to work from and report at clinics, having to work un- or under-supervised and under-equipped, having to walk long distances or depend on intermittent transport to go to people in remote areas, limit the extent, frequency and quality of community-based health care services.^23^ The model even may have given rise to new inequities because it transfers some of the cost burden of health care access to CHWs and team leaders who, in their commitment to deliver outreach, incur workload, time and out-of-pocket transport and communication expenses.

**FIGURE 1:**
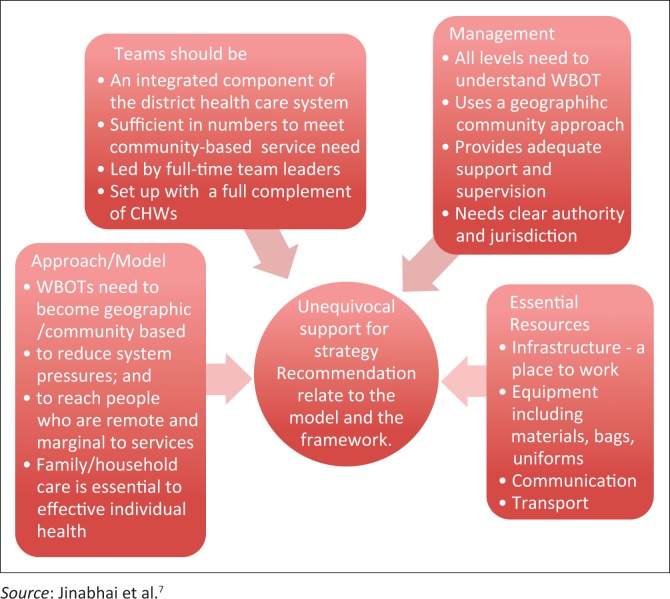
Summary analysis of themes and model recommendations.

Respondents make very clear recommendations as the summary analysis of issues and recommendations given in Figure 1 clearly shows.

These fit well with the COPC approach that is being put into practice through a City of Tshwane–University of Pretoria partnership. Implemented under the auspices of the NHI and primary care reengineering, the drive to extend universal coverage through a community-based platform has been assigned dedicated City of Tshwane funding. Located outside of facilities, teams work from local health posts with all individuals and families living and working in geographically defined spaces and places. Service and support needs therefore are defined by geographical location rather than disease. Service practices are generalist and comprehensive. Teams of CHWs are clinically led and professionally supported in their daily work by professional nurses, clinical associates, doctors and family physicians. Health care capability is developed and supported through systematic and ongoing work-integrated learning that is designed to continuously expand health literacy and build health care competency.

## Conclusions

The rapid assessment of WBOTs found extensive support for primary care reengineering at all study sites in all provinces. However, as this article has shown, respondents do not believe that a facility-based extension model is able to deliver on the promise of universal health coverage that extends to and from the home through communities, facilities and partner services.

In the South African public health context, the COPC approach to universal coverage demonstrably addresses many of the shortcomings of a facility-based extension model. This said, it continues to grapple with systemic problems of vertical programmes, poor health service integration, insufficient and under-prepared capacity, weak inter-sectoral cooperation and the marginal status of CHWs in the health care workforce.

System ambivalence makes it hard for any model to overcome vexing legacy problems as well as the intrinsic system bias towards expensive facility and specialist health care. This is especially the case in terms of financing a community health platform, securing the place of CHWs in the health care system and developing a learning system to meet the skills needs for the 21st century. In a high-stakes context laying the foundation of national wealth on a community health platform that integrates services across sectors and through systems to and from individuals and families in their homes is a low-risk strategy. Having taken the bold step of system reform through primary care reengineering, there is a need to commit to a framework and a model that will realise a strategy that is widely supported by health care providers on the front line and extensively welcomed by people in communities across the country.

## References

[1] Dahn B, Woldemariam AT, Perry H, et al. Stengthening primary care through community health workers: Investment case and financing recommendations [homepage on the Internet]. c2015 [updated 2015 Jul 15; cited 2016 Jun 30]. Available from: http://www.chwcentral.org/strengthening-primary-health-care-through-community-health-workers-investment-case-and-financing

[2] Chopra M, Lawn JE, Sanders D, et al Achieving the health millennium development goals for South Africa: Challenges and priorities. Lancet. 2009;374(9694):1023–1031. https://doi.org/10.1016/S0140-6736(09)61122-3 19709737 10.1016/S0140-6736(09)61122-3

[3] Kok MC, Dieleman M, Taegtmeyer M, et al Which intervention design factors influence performance of community health workers in low- and middle-income countries? A systematic review. Health Policy Plan. 2015;30(9):1207–1227. https://doi.org/10.1093/heapol/czu126 25500559 10.1093/heapol/czu126 PMC4597042

[4] National Department of Health National Department of Health strategic plan 2010/2011–2012/2013. Report [document on the Internet]. Pretoria: Department of Health, Republic of South Africa; 2010 [cited 2012 Jul 31]. Available from: http://www.nationalplanningcycles.org/sites/default/files/country_docs/South%20Africa/south_africa_strategic_health_plan_2010-2013.pdf

[5] Limpopo Department of Health Provincial guidelines for the implementation of the three streams of the PHC re–engineering [homepage on the Internet]. c2011 [updated 2011 Sep 4; cited 2016 Jul 01]. Available from: http://www.jphcf.co.za/wp-content/uploads/2014/06/GUIDELINES-FOR-THE-IMPLEMENTATION-OF-THE-THREE-STREAMS-OF-PHC-4-Sept-2.pdf

[6] EUROCITIES CASCADE [homepage on the Internet]. c2011 [updated 2014 May 28; 2016 Jul 03]. Available from: http://www.cascadecities.eu

[7] Jinabhai CC, Marcus TS, Chaponda A Rapid appraisal of ward-based outreach teams ASELPH(UP/FortHare) [document on the Internet]. 2015 [cited 2016 Jul 03]. Available from http://www.up.ac.za/en/family-medicine/article/2262588/copc-reports-publications

[8] Department of Health National Health Insurance in South Africa: Policy paper. Government Notice 657 of 12th August 2011. Gazette No 34523 [document on the Internet]. Pretoria; Department of Health; 2011 [cited 2016 Jul 03]. Available from: http://www.mediscor.net/docs/National%20Gazette%20No%2034523%20of%2012-August-2011,%20Volume%20554.pdf

[9] Singh P, Chokshi DA Community health workers – A local solution to a global problem. N Engl J Med. 2013;369:894–896. https://doi.org/10.1056/NEJMp1305636 24004115 10.1056/NEJMp1305636

[10] Roemer MI Evaluation of community health centres. Geneva: WHO; 1972. 4677954

[11] Bu L, Free E John B Grant international statesman of public health. Am J Public Health. 2008;98(4):628–629. https://doi.org/10.2105/AJPH.2007.129304 18309119 10.2105/AJPH.2007.129304 PMC2376987

[12] Marcus Tessa S Community oriented primary care: Origins and history. Lynnwood: Minuteman Press; 2014.

[13] Bam N, Marcus T, Hugo J, Kinkel H-F Conceptualising Community Oriented Primary Care (COPC) – The Tshwane South Africa health post model. Afr J Prim Health Care Fam Med. 2013;5(1):1–3. https://doi.org/10.4102/phcfm.v5i1.423

[14] Fryatt RJ, Andrews G, Matsoso MP, editors The South African health reforms 2009–2014: Moving towards universal coverage. Cape Town, South Africa: Juta Academic; 2015.

[15] Department of Health 2012 National health care facilities baseline audit. National summary report [homepage in the Internet]. c2012 [updated 2013 Feb; cited 2016 Jul 01]. Available from: https://www.health-e.org.za/wp-content/uploads/2013/09/National-Health-Facilities-Audit

[16] Freeman M, Hunter J, Rispel L Primary health care and universal coverage in South Africa In: Fryatt RJ, Andrews G, Matsoso MP, editors The South African health reforms 2009–2014: Moving towards universal coverage. Cape Town, South Africa: Juta Academic, 2015; p. 68.

[17] Marshall C, Whittaker S, Reddy J Improving quality In: Fryatt RJ, Andrews G, Matsoso MP, editors The South African health reforms 2009–2014: Moving towards universal coverage. Cape Town, South Africa: Juta Academic, 2015; p. 102.

[18] Global Health Workforce Alliance and World Health Organization A universal truth: No health without a workforce. Third Global Forum on Human Resources for Health Report [homepage on the Internet]. c2013 [cited 2016 Jul 01]. Available from: http://www.who.int/workforcealliance/knowledge/resources/hrhreport2013/en/

[19] Lehman U Strengthening human resources for health systems resilience to care for mothers and children. BMC Health Serv Res. 2015;15(Suppl 1):56 https://doi.org/10.1186/1472-6963-15-s1-s6 26063043 10.1186/1472-6963-15-S1-S6 PMC4464021

[20] Sidibe M, Campbell J Reversing a global health workforce crisis. Bull World Health Org. 2015;19:3 https://doi.org/10.2471/BLT.14.151209 10.2471/BLT.14.151209 PMC4271689 25558098

[21] Marcus T, Hugo J Community orientated primary care In: Mash B, editor Handbook of family medicine. 4th ed. Cape Town: Oxford University Press; 2017; p. 334–359.

[22] Schneider H, English R, Tabana H, Padayachee T, Orgill M Whole system change: Case study of factors facilitating early implementation of a primary health care reform in a South African province. BMC Health Serv Res. 2014;14:609 https://doi.org/10.1186/s12913-014-0609-y 25432243 10.1186/s12913-014-0609-y PMC4261614

[23] Mocuum R, Gomez W, Theobald S, Taegtmeyer M How equitable are community health worker programmes and which programme features influence equity of community health worker services? A systematic review. BMC Public Health. 2016;16(1):419 https://doi.org/10.1186/s12889-016-3043-8 27207151 10.1186/s12889-016-3043-8 PMC4875684

